
JOURNAL INFORMATION
==============================
NLM Title Abbreviation: ScientificWorldJournal
Journal ID: ScientificWorldJournal
NLM Journal ID: 101131163
Journal ID: TSWJ

The Scientific World Journal

ISSN: 2356-6140
EISSN: 1537-744X
Publisher: Wiley

ARTICLE INFORMATION
==============================
PMCID: PMC6084622
PMID: 30147616
DOI: 10.1100/tsw.2001.242
Article ID: 938527
Article version: 1
Subjects: Short Report

Expression Profile of Aging and Oxidative Stress Response in Drosophila melanogaster

Zou Sige *
Meadows Sarah
Sharp Linda
Jan Lily Y.
Jan Yuh Nung
Howard Hughes Medical Institute, Departments of Physiology and Biochemistry, University of California at San Francisco, San Francisco, California 94143-0725, USA
*Sige Zou: szou@itsa.ucsf.edu
Publication date: 2001
Electronic publication date: 2001 Oct 12
Volume: 1
First page: 138
Last page: 138
Copyright: Copyright © 2001 Sige Zou et al.
Copyright year: 2001
License: This is an open access article distributed under the Creative Commons Attribution License, which permits unrestricted use, distribution, and reproduction in any medium, provided the original work is properly cited.
License URL: https://creativecommons.org/licenses/by/3.0/

Funding: Howard Hughes Medical Institute

==============================
Miami *Nature Biotechnology* Short Reports

*TheScientificWorld* (2001) 1 (S3), 138SR

ISSN 1532-2246; DOI 10.1100/tsw.2001.242

### EXPRESSION PROFILE OF AGING AND OXIDATIVE STRESS RESPONSE IN *DROSOPHILA MELANOGASTER*

Sige Zou*, Sarah Meadows, Linda Sharp, Lily Y. Jan, and Yuh Nung Jan

Howard Hughes Medical Institute, Departments of Physiology and Biochemistry, University of California at San Francisco, San Francisco, California 94143-0725
* szou@itsa.ucsf.edu

**INTRODUCTION**. The free radical hypothesis of aging suggests that reactive oxygen species accumulated with age cause oxidative damages to molecules, such as nucleic acids, proteins and lipids, and are causally linked to aging and death(1). The regulation of gene expression in aging and its correlation with that in response to oxidative stress, however, is poorly understood. We have used the microarray technique to study the genome-wide expression patterns of aging and oxidative stress response in *Drosophila melanogaster*.

**METHOD**. Poly(A) isolated from fly thorax and abdomen was utilized to synthesize fluorescently labeled cDNA. For hybridization to microarrays (2), cDNA from 3-day old flies labeled with Cy3 was co-hybridized with those from 3, 10, 15, 25, 40, or 50 days old flies labeled with Cy5; cDNA from sucrose-fed flies was labeled with Cy3 and co-hybridized with cDNA from 3-hr, 12-hr, 25-hr or 34-hr paraquat-fed flies labeled with Cy5. The microarray data were submitted and stored in the AMAD database designed by J. DeRisi. The expression patterns of all the ESTs were analyzed by the Cluster and Treeview programs (3).

**RESULTS**. We measured genome-wide changes in transcript levels as a function of age for approximately 8000 ESTs represent about 4,500 genes in *Drosophila melanogaster* and compared these changes to those caused by paraquat, a free radical generator. 127 genes showed a significant change (more than 1.8 fold) in transcript levels with age and 236 genes displayed a significant change in response to paraquat. 42 genes were identified as those co-regulated with both age and paraquat treatment. Three functional groups are prominent in genes downregulated with age and oxidative stress. One includes genes encoding serine proteases and a homolog of carboxypeptidase A, suggesting that free radicals accumulated during aging reduce protein turnover. The second group includes genes encoding accessory gland protein and a homolog of Arabidopsis male sterile 2, potentially involved in reproductive capacity. The third group includes the ubiquinone binding protein, which encodes enzymes involved in energy production in mitochondria. Given that most of the free radicals in the cell are generated in the mitochondria, one way to reduce the production of free radicals might be downregulation of mitochondrial enzymes, whose activities generate free radicals. Another functional group includes genes involved in detoxification, such as arsenite-translocating ATPase and GSTD1, which are upregulated with age and oxidative stress.

**DISCUSSION**. We found that the aging process is accompanied by reduction of transcript levels for genes involved in reproduction, metabolism and protein turnover, and upregulation of some genes that encode detoxification agents and chaperones. One-third of the age-regulated genes show significant changes in response to oxidative stress, suggesting that free radicals accumulated with increasing age play a significant role in age-regulated changes in transcript levels. However, two-thirds of the age-regulated genes show no or little response to oxidative stress; free radicals therefore unlikely to be the only causal factor in aging. Further analysis of these genes could facilitate our understanding of aging and longevity at the genetic level.

**ACKNOWLEDGEMENT**. S. Z. was supported by the Damon Runyon-Walter Winchell Foundation and is now by Howard Hughes Medical Institute. L.Y.J. and Y.N.J. are HHMI investigators.

**REFERENCES.**

1. Harman, D. (1956) J. Gerontol. 2, 298-300
2. DeRisi, J.L., Lyer, V.R., and Brown, P.O. (1997) Science 278, 680-686
3. Eisen, M.B., Spellman, P.T., Brown, P.O., and Botstein, D. (1998) Proc. Natl. Acad. Sci. U S A 95, 14863-14868
